# Morphological Variations of the Human Liver: A Cadaveric Study With Clinical Correlations in a South Indian Population

**DOI:** 10.7759/cureus.105872

**Published:** 2026-03-26

**Authors:** Ramya Raju, Kalpana Ramachandran, Jayasree Srinivasan

**Affiliations:** 1 Anatomy, Sri Ramachandra Institute of Higher Education and Research, Chennai, IND

**Keywords:** accessory fissures of liver, accessory lobes of liver, clinical anatomy of liver, hepatic anatomical variations, liver morphology

## Abstract

Introduction

The liver is the largest gland and one of the most vital organs in the human body, performing essential metabolic, detoxification, and synthetic functions. Although the general anatomy of the liver is well described, morphological variations of its lobes and surfaces are frequently encountered. These variations may include accessory lobes, accessory fissures, diaphragmatic grooves, and unusual shapes of hepatic lobes. Such anatomical variations may mimic pathological lesions on imaging studies or complicate hepatobiliary surgical procedures. Therefore, understanding these variations is crucial for anatomists, radiologists, and surgeons to ensure accurate diagnosis and safe surgical planning.

Materials and methods

This cross-sectional descriptive cadaveric study was conducted on 40 human liver specimens obtained from the Department of Anatomy, Sri Ramachandra Institute of Higher Education and Research, Chennai, India. Specimens with severe pathological damage or advanced decomposition were excluded. Each liver specimen was carefully examined macroscopically to identify morphological variations in the lobes and surfaces. Variations such as accessory lobes, accessory fissures, diaphragmatic grooves, and pons hepatis were documented. Morphometric measurements, including length, width, and depth of accessory fissures and grooves, were recorded using standard measuring instruments. The observations were analyzed and presented as frequencies and percentages.

Results

Morphological variations were observed in 19 (47.5%) of the examined specimens. The left lobe demonstrated the most frequent variation, with a tongue-like projection present in 16 (40%) of specimens. Variations of the right lobe were observed in 6 (15%) of cases, including deep renal impressions, hypoplastic right lobe, elongated right lobe, and accessory lobes. The caudate lobe showed variations in shape, including rectangular, bilobed, and pyriform. Quadrate lobe variations were relatively uncommon and noted in 3 (7.5%) of specimens. Accessory fissures were frequently observed, particularly in the right lobe (23; 57.5%). Diaphragmatic grooves were present in 13 (32.5%) of specimens, while pons hepatis was identified in 9 (22.5%) of cases.

Conclusion

Morphological variations of the liver are relatively common and may significantly influence radiological interpretation and hepatobiliary surgical procedures. Awareness of these variations is essential for accurate diagnosis and effective surgical planning, thereby reducing the risk of misinterpretation and intraoperative complications.

## Introduction

The liver, the largest gland and the second-largest solid organ in the human body, performs a variety of functions, such as metabolism, synthesis, and detoxification, that are crucial for homeostasis [1,2]. It is located primarily in the right hypochondrium and in the epigastric regions of the abdomen. It also partly extends into the left hypochondrium, retaining its location predominantly under the right hemidiaphragm. The division of the liver anatomically into a greater part (the right lobe) and a smaller part (the left lobe) is marked by the peritoneal attachment of the falciform ligament in the anterior aspect and by the fissure for the ligamentum venosum and teres in the posterior and inferior aspects [3]. The liver further features the caudate lobe and the quadrate lobe, which are considered part of the right lobe anatomically. However, the caudate and quadrate lobes are functionally distinct and are demarcated by the location of the porta hepatis in the liver [4].

Although the standard lobar anatomy is well documented, morphological variations of the human liver are remarkably common and manifest as either congenital anomalies or acquired structural modifications [5]. Congenital variations often result from aberrant embryonic development, presenting as complete agenesis, hypoplasia, or marked atrophy of specific lobes [3]. Furthermore, the hepatic morphology may be significantly altered by the presence of accessory fissures and accessory lobes, the most clinically recognized of the latter being Riedel’s lobe. Additional structural variations include atypical configurations of the caudate and quadrate lobes, such as a prominent papillary process, a bilobed appearance, or the presence of a pons hepatis bridging the umbilical fissure, alongside deep diaphragmatic grooves on the superior surface [5].

The clinical recognition of these morphological variations is of vital importance in modern clinical practice, especially in fields concerned with hepatobiliary surgery, liver transplantations with living donors, and laparoscopic procedures that are minimally invasive [6]. In the world of diagnostic imaging, with modalities such as computed tomography (CT), magnetic resonance imaging (MRI), and ultrasonography (USG), the above-mentioned morphological variations can often lead to misinterpretations or diagnostic errors [5]. For example, any fluid accumulating within the accessory fissures may be easily misdiagnosed as a case of intrahepatic hematoma, a liver abscess, or a laceration following abdominal trauma [5]. In a similar manner, because accessory lobes are often associated with a vascular pedicle, they might be mistaken for lesser omental lymphadenopathy or a case of metastatic tumor. A correct identification of such accessory lobes becomes crucial because they carry a significant risk of torsion or infarction and may lead to fatal hemorrhagic complications during surgery [3].

Despite the remarkable advancements in noninvasive imaging modalities capable of yielding high-resolution images, a detailed macroscopic inspection and evaluation of the cadaveric liver specimens remains an essential and incomparable method for documentation of these intricate variations. The prevalence and structural complexity of these hepatic anomalies are clearly interpreted by the study of cadaveric specimens, which provides us with direct, accurate physical evidence [2]. This evidence is essential for fine-tuning anatomical knowledge, which provides the foundation of medical education, radiological diagnosis, and preoperative planning [7].

Therefore, the objective of the present study is to systematically inspect and document the gross morphological variations of the liver that are observed in cadaveric liver specimens, while giving a detailed account of the presence of accessory lobes, fissures, and lobar anomalies. By providing a comprehensive map of these structural variations, this study hopes to provide insight into their clinical and anatomical significance. The findings from the study aim to assist hepatobiliary surgeons, radiologists, and gastroenterologists in enhancing diagnostic accuracy and minimizing surgical morbidity.

## Materials and methods

Primary and secondary objectives

This study primarily aims to evaluate and document the gross morphological variations of the human liver in cadaveric specimens. The secondary objectives are the following: (1) to assess the frequency and distribution of accessory lobes, fissures, and lobar variations; (2) to perform morphometric analysis of identified variations; (3) to analyze correlations between morphometric parameters; and (4) to discuss the clinical implications of these variations in radiological and surgical practice.

This study was undertaken in the Department of Anatomy at Sri Ramachandra Institute of Higher Education and Research (SRIHER), Tamil Nadu, India. The Institutional Ethics Committee (for Medical PG Students), SRIHER (DU), issued approval (approval no. CSP/MED/24/SEP/109/322).

Specimen selection

A periodic sampling approach was used for the study. Over the course of the one-year study period, a representative set of 40 human liver specimens was screened using the exclusion criteria.

Exclusion criteria

Specimens showing significant putrefaction or morphological disruption were excluded from the study. Any cadaveric liver specimens presenting with gross pathology such as cirrhosis, cysts, or carcinoma were excluded. This was done to avoid confounding our results with these structural changes. Following the application of exclusion criteria, 40 cadaveric liver specimens were used for the purpose of the study and were selected using the periodic sampling method.

Morphological and morphometric assessment

Each specimen was systematically inspected and examined macroscopically to identify gross anatomical variations. Special attention was given to the following: (1) lobar anatomy: the number of lobes present was recorded, and the absence of any lobes or the presence of any additional lobes was noted. (2) Surface and border characteristics: the hepatic surfaces and borders were carefully inspected for the presence of any gross anatomical variations. (3) Congenital and rare variants: the specimens were also screened for the presence of any congenital or rare variants, such as Riedel’s lobe, pons hepatis, or congenital hepatic fibrosis.

The quantitative perspectives of the anatomical variations were given by the measurements of length, breadth, and depth of accessory fissures, diaphragmatic grooves, accessory lobes, pons hepatis, and Riedel’s lobe, wherever applicable. The measurements were taken using a measuring scale.

Data management and statistical analysis

The quantitative and qualitative data so obtained were tabulated in Microsoft Excel 2019 (Microsoft Corporation, Redmond, Washington) and statistically analyzed using IBM SPSS Statistics for Windows, Version 23 (Released 2015; IBM Corp., Armonk, New York). Qualitative variables were expressed as frequencies with percentages. Quantitative variables were expressed as either means with standard deviations or medians with interquartile ranges based on the normality testing of the data. A Spearman's correlation analysis was done between the width and the breadth of the grooves. A *p*-value of less than 0.05 was considered statistically significant.

## Results

Specimen demographics

Among the 40 cadaveric liver specimens examined for the purpose of the study, morphological variations were identified in 19 (47.5%) specimens. Neither the right lobe nor the quadrate lobe of the liver specimens exhibited many variations, so the highest frequency of variations was found in the left lobe and the caudate lobe of the liver specimens. Table 1 presents a detailed frequency distribution of morphological variations observed in the liver specimens in the present study.

**Table 1:** Frequency distribution of morphological variations of the liver (n = 40)

Lobes	Variations	Frequency	Percentage (%)
Right Lobe	Lengthier	1	2.50%
	Deep renal impression	2	5.00%
	Small	2	5.00%
	Small and accessory lobe	1	2.50%
	Normal	34	85.00%
Left Lobe	Tongue-like projection	16	40.00%
	Butterfly-shaped	1	2.50%
	Elongated left lobe	1	2.50%
	Flat	1	2.50%
	Small	2	5.00%
	Normal	19	47.50%
Caudate Lobe	Normal	21	52.50%
	Bilobed	5	12.50%
	Hypertrophied	1	2.50%
	Pyriform	5	12.50%
	Rectangular caudate process present and papillary process absent	1	2.50%
	Rectangular	6	15.00%
	Small	1	2.50%
Quadrate Lobe	Normal	37	92.50%
	Accessory lobe	2	5.00%
	Rectangular	1	2.50%

Lobe-specific morphological variations

Right and Left Lobes

The right lobe was found to exhibit variations in only 6 (15%) of the specimens examined. The variations encountered included deep renal impressions in 2 (5%) specimens and a small or hypoplastic right lobe in 2 (5%) specimens. The other variations were the right lobe being long in 1 (2.5%) specimen and the right lobe being small, along with the presence of an accessory lobe in 1 (2.5%) specimen. Conversely, the left lobe demonstrated significant morphological variability, with only 19 (47.50%) exhibiting standard anatomical features. The most commonly observed variation was a tongue-like projection that was present in 16 (40%) of the specimens. The other variations observed in the left lobe of the liver included a butterfly-shaped lobe in 1 (2.5%), an elongated left lobe in 1 (2.5%), a flat left lobe in 1 (2.5%), and a small or hypoplastic left lobe in 2 (5%) specimens.

Caudate and Quadrate Lobes

**Figure 1:**
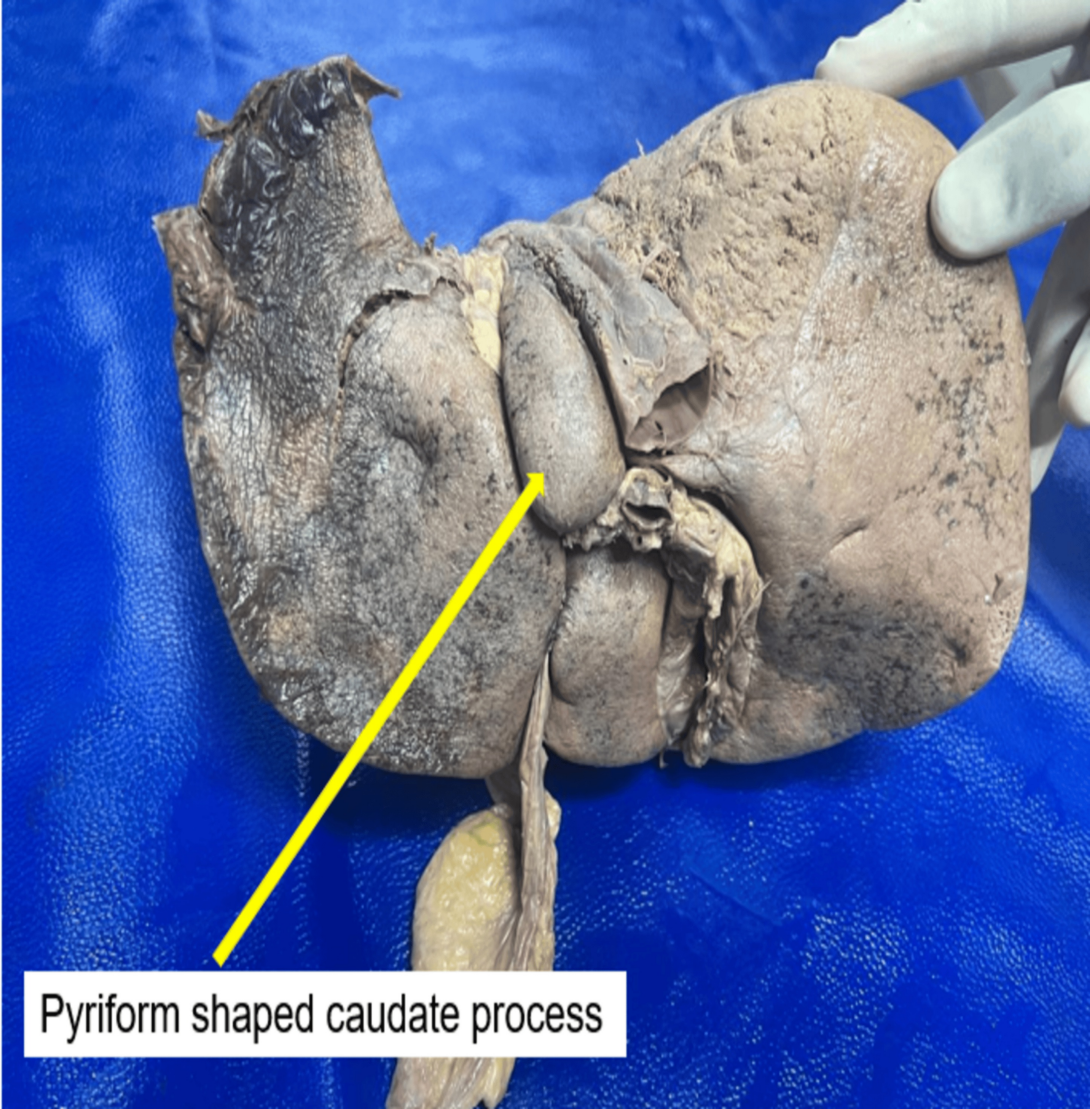
Pyriform caudate process (yellow arrow)

A normal morphology of the caudate lobe was noted in 21 (52.5%) of the examined liver specimens. The observed variations of the caudate lobe included a rectangular shape of the lobe in 6 (15%) of the specimens, a bilobed caudate lobe in 5 (12.5%) of the specimens, and a pyriform caudate lobe in 5 (12.5%) of the specimens. The quadrate lobe exhibited variations in a minority of 3 (7.5%) of the liver specimens examined. The most common variation observed in the quadrate lobe was the presence of an accessory lobe in 2 (5%) of the specimens (Figure 1).

Normality testing

To determine the appropriate statistical expression, the Shapiro-Wilk test was conducted on all morphometric parameters. All measured variables, including the dimensions of diaphragmatic grooves, followed a non-normal distribution (p < 0.005). Therefore, the morphometric data are expressed as medians with interquartile ranges.

Accessory fissures

**Figure 2:**
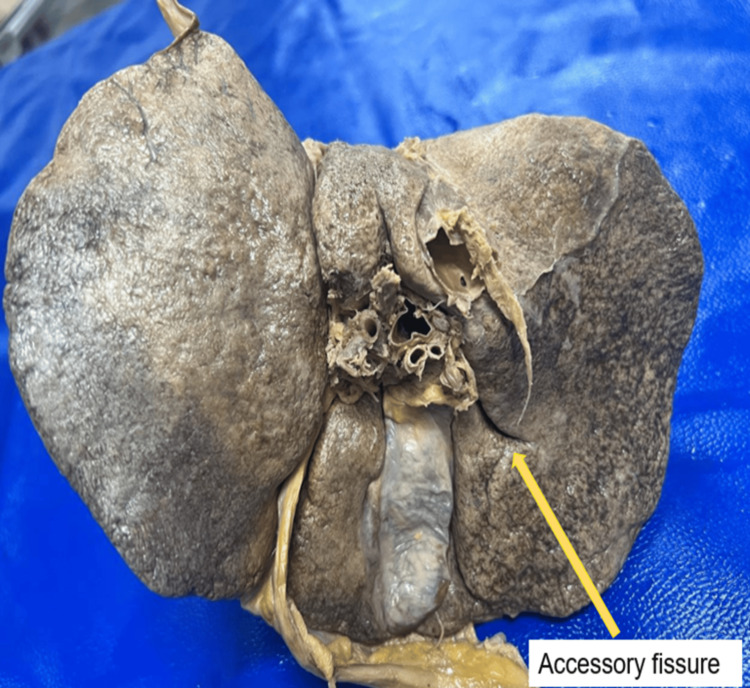
Accessory fissure (yellow arrow)

Accessory fissures were most frequently present in the right lobe of the 23 (57.5%) specimens (Figure 2). Table 2 provides a detailed list of the frequency distribution of accessory fissures. Right lobe: Among the 23 (57.5%) specimens in which accessory fissures were observed in the right lobe, 15 (37.5%) specimens had a single accessory fissure, and 8 (20%) specimens had two accessory fissures. The accessory fissures in the right lobe had a median length of 2.50 cm (1.60-5.40 cm), a width of 0.25 cm (0.20-0.50 cm), and a depth of 0.50 cm (0.35-1.00 cm). Left lobe: Among the 40 specimens examined, 2 (5%) demonstrated the presence of an accessory fissure in the left lobe. These accessory fissures had a median length of 2.75 cm, a width of 0.35 cm, and a depth of 0.65 cm.

**Table 2:** Frequency distribution of accessory fissures (n = 40)

Lobes in the Liver	Variations	Frequency	Percentage (%)
Right Lobe	One	15	37.50%
	Two	8	20.00%
	Normal	17	42.50%
Left Lobe	One	2	5.00%
	Normal	38	95.00%

Diaphragmatic grooves

**Figure 3:**
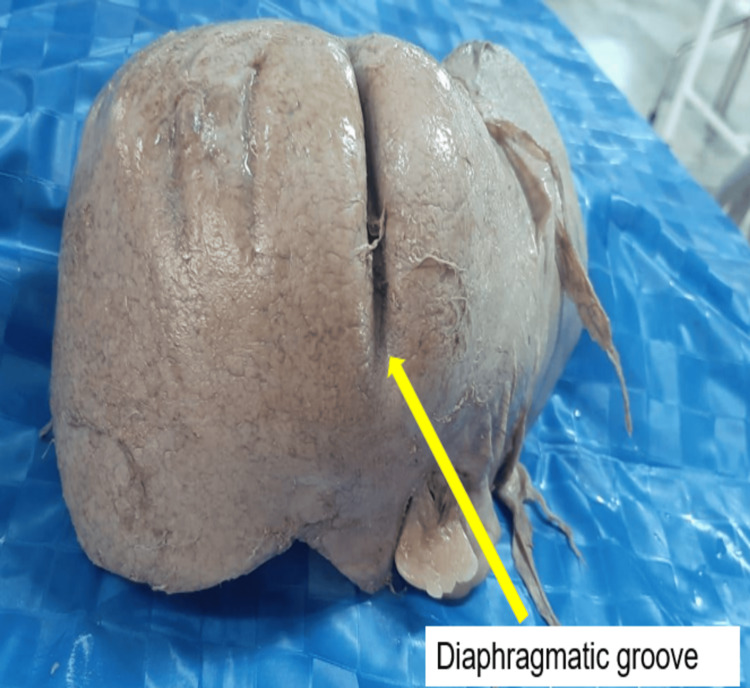
Diaphragmatic groove (yellow arrow)

**Figure 4:**
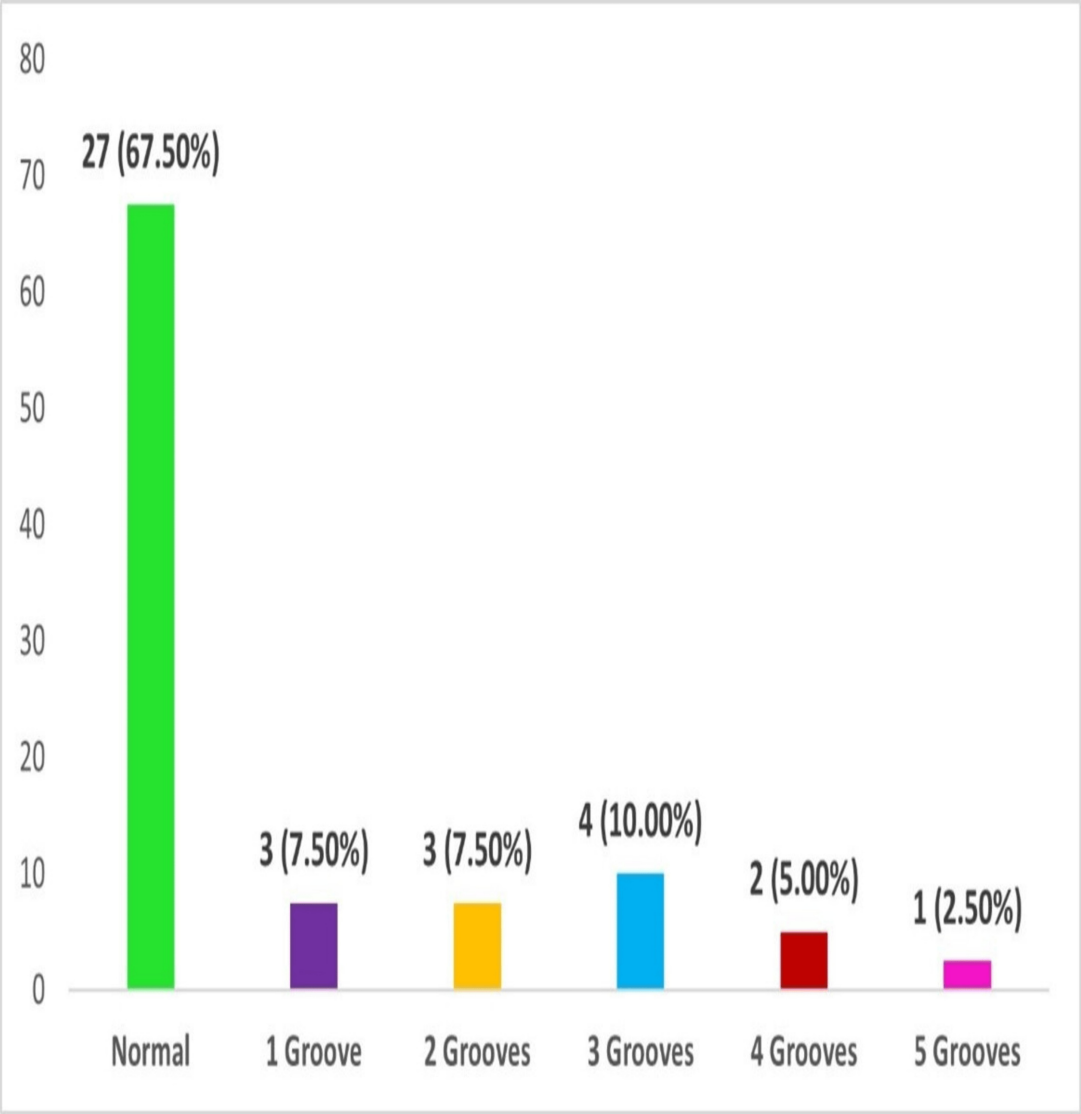
Frequency distribution of bar graphs per specimen

Diaphragmatic grooves were observed in 13 (32.5%) of the total specimens examined. The number of grooves present in each specimen ranged from 1 to 5 (Figure 3). Dimensions: The median length of the groove was 4.50 cm (3.00-6.50); the width was 0.40 cm (0.20-0.80); and the depth was 0.20 cm (0.10-0.40). One specimen had a diaphragmatic groove with a maximum length of 14.0 cm (Figure 4).

Correlation analysis (Spearman’s rank correlation)

**Figure 5:**
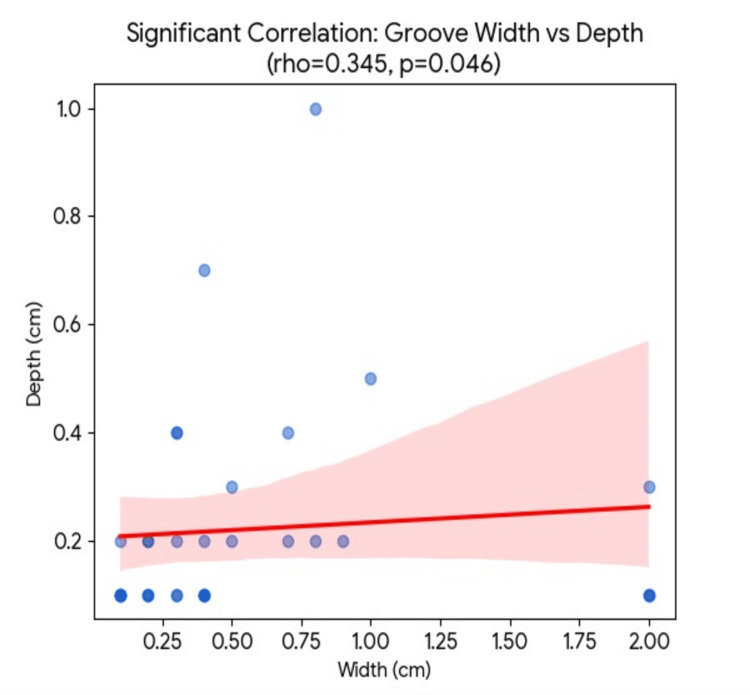
Spearman’s rank correlation

Spearman’s rank correlation (rho) was used to evaluate the relationships between the dimensions of the observed anatomical variations. Dimensional proportionality: Within the diaphragmatic grooves, a statistically significant positive correlation was identified between the width and the depth of the grooves (rho = 0.345, p = 0.0459), indicating that wider grooves tend to be deeper within the hepatic parenchyma. Fissural dimensions: For the accessory fissures observed in the right lobe, the correlation between the length and the depth of the fissure was moderate and was near the statistically significant range (rho = 0.350, p = 0.0631). No significant correlation was found between the number of diaphragmatic grooves and the number of accessory fissures per specimen (rho = -0.028, p = 0.863), suggesting that these features have an independent development (Figure 5).

Minor variations

**Figure 6:**
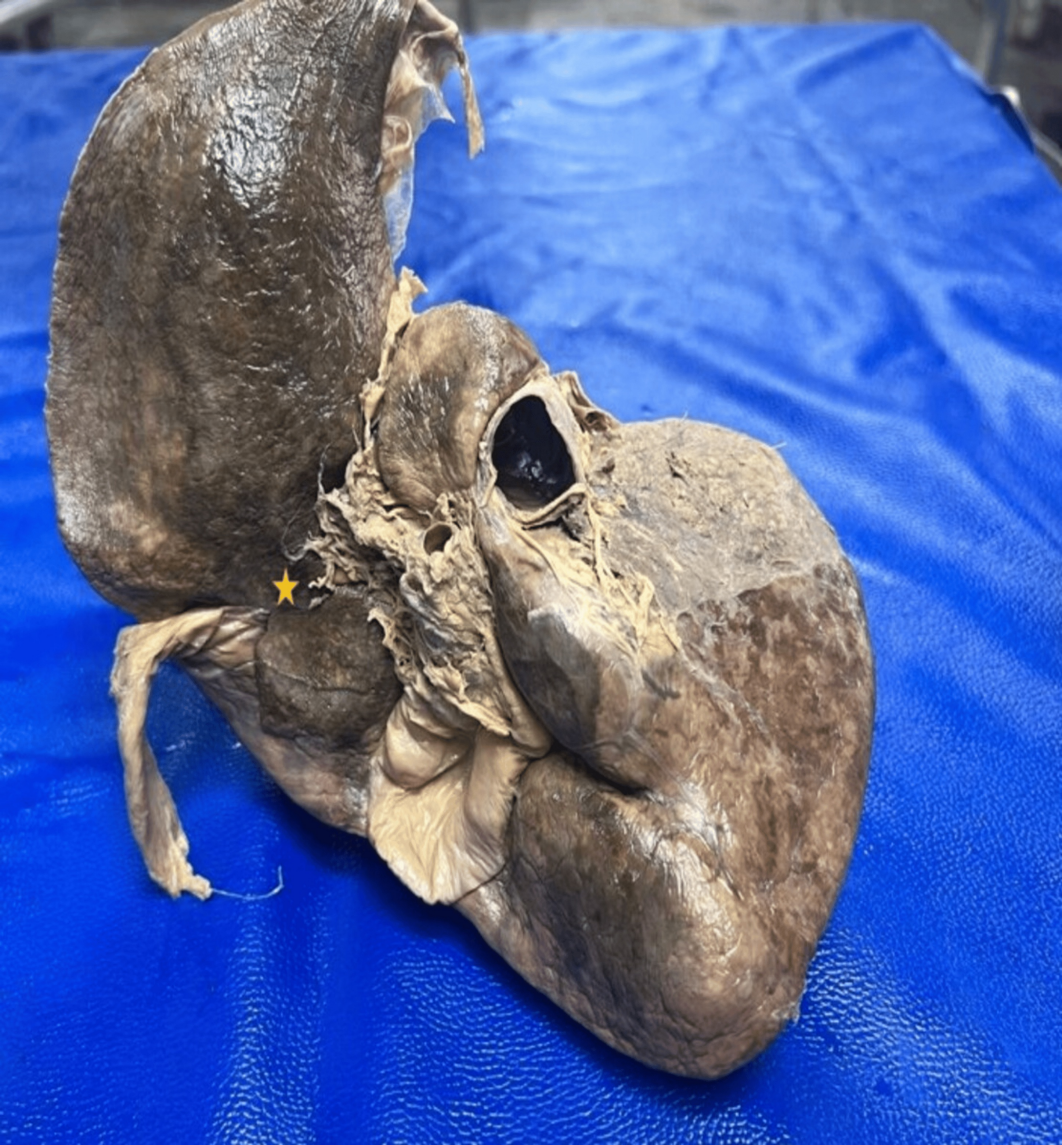
Pons hepatis (yellow star)

**Figure 7:**
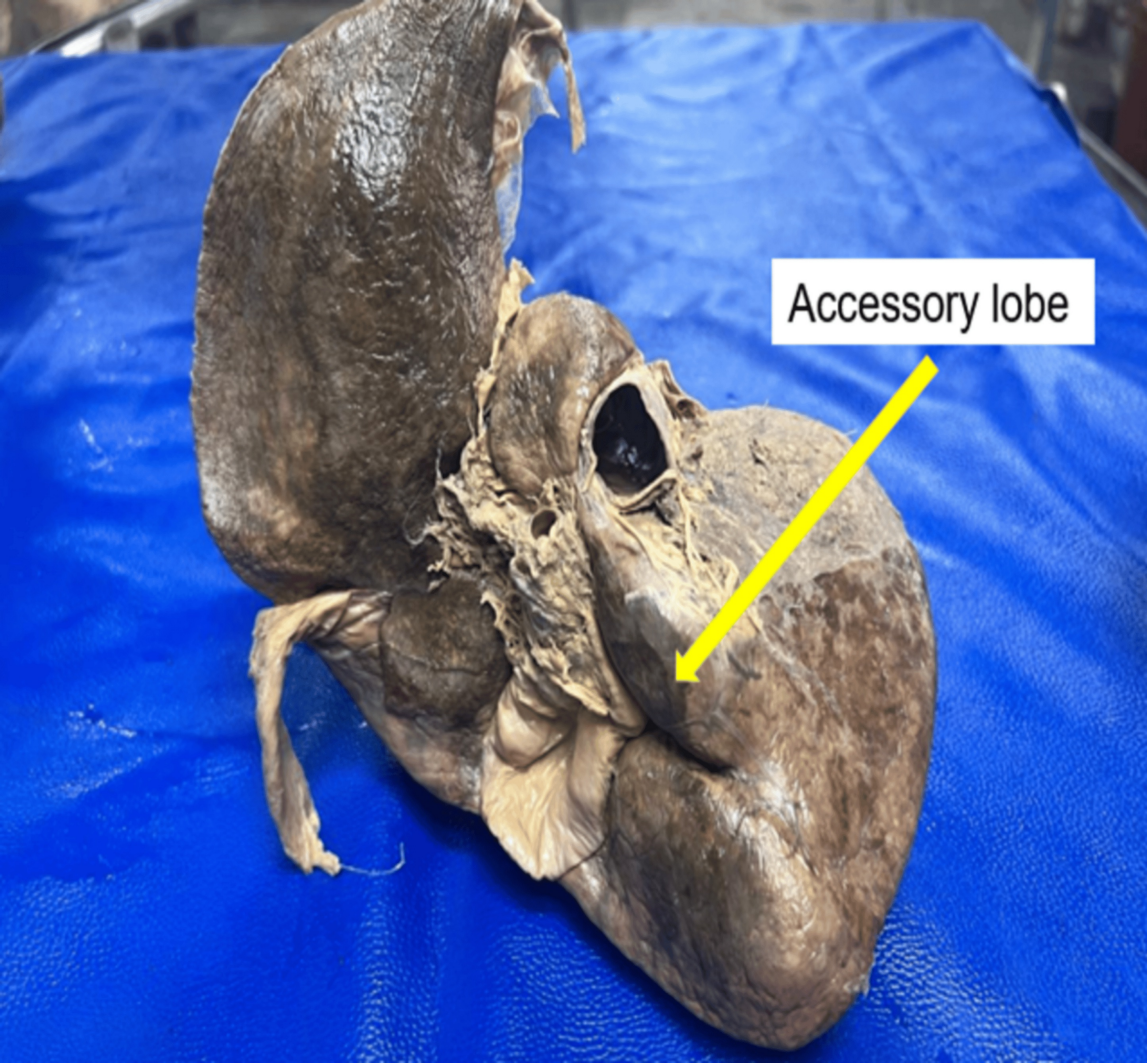
Accessory lobe (yellow arrow)

**Figure 8:**
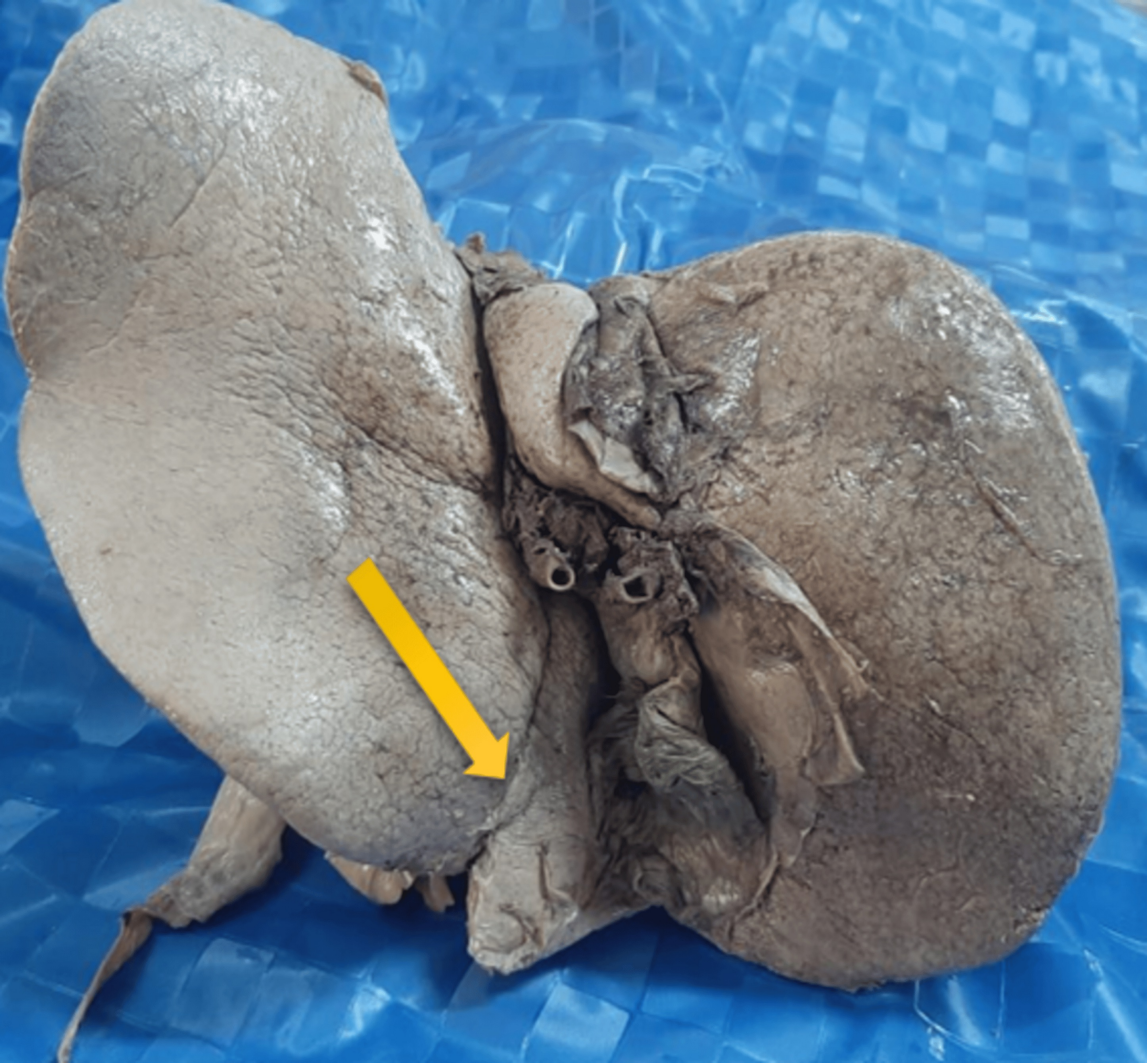
Absent fissure for ligamentum teres (yellow arrow)

Pons hepatis was present in 9 (22.5%) specimens, effectively bridging the fissure for ligamentum teres. The median bridge length was 1.85 cm (1.40-2.00) (Figure 6). Accessory lobes were identified in 1 (2.5%) of the left lobes, measuring 2.9 cm x 2.5 cm (Figure 7). Ligamentum teres fissure: Widening of this fissure occurred in 7 (17.5%) of cases (Figure 8). Riedel’s lobe: This variation was entirely absent across the 40-specimen cohort.

## Discussion

The current study evaluated morphological variations in 40 cadaveric livers and found that structural abnormalities were present in 19 specimens (47.5%). Notably, right-sided accessory fissures were a common feature in 23 (57.5%) of specimens, and prominent diaphragmatic grooves were observed in 13 (32.5%) of specimens, with a significant positive correlation between groove width and depth. The absence of Riedel's lobe, a 1 (2.5%) specimen incidence of accessory lobes, and a 9 (22.5%) prevalence of pons hepatis further emphasize the macroscopic diversity of human hepatic anatomy.

Embryological correlation

The intricate processes of early embryonic development are the source of the morphological variations of the human liver seen in this study. The hepatic diverticulum, also known as the liver bud, appears from the endodermal foregut during the third week of pregnancy and quickly multiplies into the mesoderm of the septum transversum. The persistence of embryonic mesodermal septa that do not entirely regress during this parenchymal expansion is usually the cause of accessory fissures [8]. The mechanical resistance of hypertrophied diaphragmatic muscle bundles pressing against the quickly expanding, pliable hepatic parenchyma during fetal life produces diaphragmatic grooves, which eventually leave deep impressions [4]. Additionally, an abnormal, excessive proliferation of hepatoblasts over the umbilical vein within the falciform ligament causes the formation of a pons hepatis, which bridges the quadrate and left lobes. Similarly, accessory lobes form when a portion of the primitive hepatic rudiment is completely displaced or pinched off by the surrounding mesodermal tissue, separating from the main hepatic mass while maintaining a distinct vascular pedicle [9].

Comparison with previous literature

Seeja et al. found that of their 50-specimen cohort, 14 (28%) had accessory fissures in the right lobe and 4 (8%) had diaphragmatic grooves [10]. These results are inconsistent with those of the current study, which found a significantly higher prevalence of diaphragmatic grooves (13; 32.5%) and right lobe accessory fissures (23; 57%).

Sambhav et al. found that diaphragmatic grooves were present in 3 (7.5%) of livers and that right lobe accessory fissures were present in a staggering 29 (72.5%) of livers [8]. The remarkably high presence of accessory fissure rate is somewhat consistent with the strong 23 (57.5%) prevalence of specimens found in the right lobe specimens of the current study. Our results, however, show that 13 (32.5%) of specimens had diaphragmatic grooves, which contrasts with their low rate.

Chauhan et al. found diaphragmatic grooves in 13 (25%) of the specimens examined and pons hepatis bridging the umbilical fissure in 5 (9.61%) of the liver specimens [5]. These findings are in strong agreement with the present study, which reports pons hepatis in 9 (22.5%) of specimens and diaphragmatic grooves in 13 (32.5%) of the examined specimens. The identification of the pons hepatis is crucial for hepatobiliary surgeons because it obscures anatomical landmarks and makes it difficult to delineate the left hepatic boundary during segmental resections [5].

Deshwal et al. found a 7 (20%) prevalence of diaphragmatic grooves in their study and a 12 (34.28%) prevalence of accessory fissures [4]. These results are somewhat consistent with the present study, even though the present study found a common occurrence of accessory fissures in the right lobe.

Anbumani et al. found that 4 (13.3%) of the 30 liver specimens examined had pons hepatis, 6 (20%) had diaphragmatic fissures, and 12 (40%) had accessory fissures in the right lobe [9]. Their results are highly consistent with the present study, which also found that the accessory fissures were most commonly found in the right lobe. The present study also reports a significantly high percentage of diaphragmatic grooves (13; 32.5%) and pons hepatis (9; 22.5%).

In contrast, a study conducted by Chaudhari et al. reported a 10 (12.5%) occurrence of accessory fissures, a 6 (7.5%) presence of diaphragmatic grooves, and a 1 (1.25%) presence of pons hepatis out of the 80 liver specimens examined for the study purpose [3]. These findings are in stark contrast to those of the present study, which report a 9 (22.5%) prevalence of pons hepatis and a 23 (57.5%) presence of accessory fissures in the right lobe.

In a study conducted by Kaur et al., which examined 32 liver specimens for the presence of morphological variations, the accessory fissures were found to be in 18 (56.25%) of the specimens and pons hepatis in 6 (18.75%) of the specimens. These prevalence rates of accessory fissures and pons hepatis are consistent with the present study’s findings. Their study also reinforced the significance of identifying these parenchymal bridges because they may pose serious challenges during surgery [11].

Bhardwaj et al. examined a large cohort of 110 specimens to understand the morphological variations of the liver. They reported a 37 (33.63%) prevalence of diaphragmatic sulci and accessory fissures. The current study also reports a combined high prevalence of diaphragmatic grooves and accessory fissures, which is consistent with their data. Bhardwaj et al. attributed the high prevalence of these diaphragmatic sulci to the mechanical pressure exerted by the diaphragm [12]. The present study’s significant statistical correlation between the width and depth of the diaphragmatic groove provides strong evidence to support this mechanical hypothesis.

Ramachandran et al. found a high prevalence of accessory lobes (9; 25.7%) and accessory fissures (13; 37.1%) of the specimens examined [13]. These results are in contradiction to the present study, which found a lower prevalence rate of accessory lobes in 1 (2.5%) specimen and a much higher prevalence rate of accessory fissures (23; 57.5%) in the right lobe.

A high prevalence of accessory lobes (6; 12%) and accessory fissures (5; 10%) was found in a study conducted by Khajuria et al. [14]. These findings contradict those of the present study, which report a higher prevalence of accessory fissures in 23 (57.5%) specimens and a significantly lower rate of accessory lobes in 1 (2.5%) specimen.

Anasuya et al. reported a 20 (40%) occurrence of accessory fissures, a 14 (28%) occurrence of diaphragmatic grooves, and a 9 (18%) presence of pons hepatis [6]. These findings correlate with the present study, with observations of a 23 (57.5%), 13 (32.5%), and 9 (22.5%) specimen occurrence of accessory fissures, diaphragmatic grooves, and pons hepatis, respectively.

In a study focused on the caudate lobe conducted by Deepa et al., the incidence of a rectangular shape of the lobe was reported in 22 (44%) specimens, a pear-shaped caudate lobe was seen in 17 (34%) specimens, 7 (14%) specimens were bicornuate, and 11 (22%) specimens were considered irregular [15]. The present study, which also reports a high frequency of morphological variations in the caudate lobe, partially concurs.

Bedi et al. found that the quadrate and the left lobes fused together, forming the pons hepatis, in 4 (10%) of the specimens, and diaphragmatic grooves were present in 9 (22.5%) of the specimens [1]. Their results are highly consistent with the present study, which reports a 9 (22.5%) incidence of pons hepatis and a 13 (32.5%) incidence of diaphragmatic grooves.

Srivastava et al. (2020) noted that variant lobes preserved normal internal histological architecture and reported a 28 (71.79%) rate of normal gross morphology [2]. This is somewhat consistent with the current study, which discovered a variety of morphological variations that functioned as nonpathological adaptations at a higher gross rate of 19 (47.5%) specimens. The accessory lobes, pons hepatis, and deep fissures seen in our cohort were benign, functional physiological variations rather than dysplastic or premalignant malformations.

The significance of accessory lobes, their attachment through a vascular pedicle, and proximity to the porta hepatis were highlighted in a study conducted by Sawant et al., who found accessory lobes to be found in 5 (10%) of their cadaveric specimens [16]. The present study, however, reports a significantly lower incidence of accessory lobes in 1 specimen (2.5%).

Thorat et al. found that diaphragmatic grooves accounted for 25 (15.6%) of the anomalies, whereas 83 (51.9%) of their 160 specimens showed no morphological variations [17]. This is consistent with the current study, which found that although our specific rate of diaphragmatic grooves was higher (13; 32.5%), a comparable 21 (52.5%) of specimens were morphologically normal.

Regina et al. found that 3 (6%) of the cohort had diaphragmatic grooves and 6 (12%) had accessory fissures on the right lobe [7]. Their results support the frequent occurrence of these variations, but they are at odds with the current study, which found much higher frequencies of diaphragmatic grooves (13; 32.5%) and right lobe fissures (23; 57.5%).

Sangeetha et al. found that 12 (24%) of their specimens had accessory fissures, 10 (20%) had diaphragmatic grooves, and 3 (6%) had pons hepatis [18]. The current study shows significantly higher incidences of diaphragmatic grooves in 13 (32.5%), pons hepatis in 9 (22.5%), and right lobe accessory fissures in 23 (57.5%) specimens, but it also partially agrees with the types of variations found.

Srimani et al. found that 78 (70.9%) of their sample had variant livers, with a 39 (35.5%) incidence of pons hepatis, a 12 (10.9%) incidence of diaphragmatic grooves, and a 54 (49.1%) incidence of extensive accessory fissures on the right lobe [19]. The results of the current study, which showed a high overall variation rate, a significant prevalence of pons hepatis in 9 (22.5%) specimens, and robust accessory fissures, are strongly supported by this finding.

Ragavan et al. found diaphragmatic sulci in 18 (19.35%) of livers, pons hepatis in 21 (22.5%), and accessory fissures in 48 (51.61%) of livers [20]. These findings are highly consistent with the current study, which found pons hepatis in precisely 9 (22.5%) of the specimens and right lobe accessory fissures in 23 (57.5%).

Table 3 summarizes the present study's findings and compares them with previous research.

**Table 3:** Comparison of the present study findings with previous literature

Study	Diaphragmatic Grooves n (%)	Accessory Fissures n (%)	Pons Hepatis n (%)	Accessory Lobes n (%)	Other Variations
Current Study	13 (32.5)	23 (57.5)	9 (22.5)	1 (2.5)	19 (47.5) variant morphology of the liver
Ramachandran et al. [13]	-	13 (37.1)	-	9 (25.7)	-
Khajuria et al. [14]	-	5 (10)	-	6 (12)	-
Sawant et al. [16]	-	-	-	5 (10)	Attachment through the vascular pedicle
Bhardwaj et al. [12]	37 (33.63)	37 (33.63)	-	-	Prevalence reported as combined for sulci and fissures
Srimani et al. [19]	12 (10.9)	54 (49.1)	39 (35.5)	-	70.9% variant livers
Anbumani et al. [9]	6 (20)	12 (40)	4 (13.3)	-	-
Anasuya Geeta et al. [6]	14 (28)	20 (40)	9 (18)	-	-
Ragavan et al. [20]	18 (19.35)	48 (51.61)	21 (22.5)	-	-
Kaur et al. [11]	-	18 (56.25)	6 (18.75)	-	-
Sambhav et al. [8]	3 (7.5)	29 (72.5)	-	-	-
Sangeetha et al. [18]	10 (20)	12 (24)	3 (6)	-	-
Chaudhari et al. [3]	6 (7.5)	10 (12.5)	1 (1.25)	-	-
Seeja et al. [10]	4 (8)	14 (28)	-	-	-
Deshwal et al. [4]	7 (20)	12 (34.28)	-	-	-
Chauhan et al. [5]	13 (25)	-	5 (9.61)	-	-
Bedi et al. [1]	9 (22.5)	-	4 (10)	-	-
Thorat et al. [17]	25 (15.6)	-	-	-	83 (51.9) no morphological variations
Regina et al. [7]	3 (6)	6 (12)	-	-	-
Deepa et al. [15]	-	-	-	-	Rectangular shape of caudate lobe in 22 (44%), pear-shaped caudate lobe 17 (34%), bicornuate 7 (14%), irregular 11 (22%)

Clinical significance

Having a thorough understanding of these morphological variations is crucial to the prevention of errors in diagnosis and surgical complications. Accessory fissures often lead to misdiagnosis during radiological interpretation. Fluid accumulation within these accessory fissures can be falsely interpreted as liver cysts, intrahepatic hematomas, liver abscesses, or lacerations following abdominal trauma. These fissures can also act as spaces for the implantation of tumor cells that mimic macronodular cirrhosis or intrahepatic focal lesions [21]. Conversely, certain accessory fissures like the Rouvière's sulcus may also prove useful by serving as vital landmarks for the identification of biliary pedicles during the procedure of laparoscopic cholecystectomy [18].

Smaller accessory lobes located near the porta hepatis may often be falsely concluded as lesser omental lymphadenopathy and be inadvertently damaged, leading to severe hemorrhage. Because accessory lobes are often associated with vascular pedicles, they inherently carry a risk of torsion, leading to infarction and rupture. These conditions often require emergency surgical interventions [6].

Any variations in the anatomical right and left lobar sizes also carry diagnostic significance. Hypoplasia of the left lobe is often associated with gastric volvulus, whereas hypoplasia of the right lobe may progress to portal hypertension [5]. In contrast, elongation of the left lobe or the presence of a tongue-like projection may present with epigastric pain [21].

A pons hepatis bridging the quadrate and left lobe conceals the fissure for ligamentum teres, often causing complications in surgical navigation [9].

Limitations

Although the study provides a detailed morphological and morphometric analysis of variations of the liver, several limitations should be considered. The study was performed on a relatively small sample size cohort of 40 liver specimens. The study was conducted on embalmed liver specimens, which could have caused alteration in the measurement of these variations due to hardening of the tissues. Because the study was performed on cadaveric liver specimens, demographic data such as age, gender, and physical stature were not available for comparison with the observed variations. The findings of the present study are present on naked-eye inspection and also lack interobserver variability and a dynamic perspective of in vivo radiological imaging.

Future direction

In the future, larger studies with radiological correlation, interobserver assessment, and demographic stratification can be done to further validate and extend our findings.

## Conclusions

The current study demonstrates that the liver’s morphological variations, such as pons hepatis, diaphragmatic grooves, and accessory fissures, are extremely common and harmless anatomical adaptations. Significant population and methodological variability is revealed by comparisons with the literature, especially regarding the precise prevalence of surface clefts and accessory lobes. There is general agreement that these abnormalities result from clearly defined embryological deviations involving mesodermal persistence and uneven parenchymal proliferation, despite these numerical variations. To prevent misinterpreting physiological fissures as traumatic lacerations or metastatic deposits on radiological imaging, it is crucial from a clinical standpoint to identify these variations. In the end, a thorough macroscopic understanding of these hepatic variations enables hepatobiliary surgeons to safely navigate through abnormal vascular and biliary pedicles, greatly lowering the risk of iatrogenic complications during difficult resections and transplants.
